# Equivalent Circuit Model Extraction for a SAW Resonator: Below and above Room Temperature

**DOI:** 10.3390/s22072546

**Published:** 2022-03-26

**Authors:** Giovanni Gugliandolo, Zlatica Marinković, Giovanni Crupi, Giuseppe Campobello, Nicola Donato

**Affiliations:** 1 Department of Engineering, University of Messina, 98158 Messina, Italy; giuseppe.campobello@unime.it (G.C.); ndonato@unime.it (N.D.); 2 Faculty of Electronic Engineering, University of Niš, 18000 Niš, Serbia; zlatica.marinkovic@elfak.ni.ac.rs; 3 BIOMORF Department, University of Messina, 98125 Messina, Italy; crupig@unime.it

**Keywords:** surface acoustic wave resonators, resonance, electrical characterization, admittance parameters, temperature, curve fitting, Lorentzian fitting

## Abstract

In this work, a SAW resonator is characterized in terms of admittance (*Y*-) parameters in the temperature range spanning from 0 °C to 100 °C, with the aim of highlighting how its physical properties are affected by the temperature change. A lumped-element equivalent-circuit model is used to represent the device under test at the considered temperature conditions and a parameters extraction process based on a Lorentzian fitting is developed for the determination of the equivalent-circuit elements in the investigated temperature range. A very good agreement is observed between the performed measurements and the model simulations. The characterization process and the subsequent equivalent-circuit parameters extraction at different temperature values are described and discussed.

## 1. Introduction

Surface acoustic wave (SAW) devices today represent a hot research topic because of their widespread use in several fields. They are compact, easy to fabricate, and cost-effective. The concept of SAW technology was first explored in 1885 by Lord Rayleigh, who studied the propagation of acoustic waves in piezoelectric materials [1]. The first SAW device was presented by White and Voltmer in 1965 [2]. They proposed a way to generate a surface acoustic wave by using interdigitated electrodes. However, because of the complicated fabrication steps, not much interest was given to this emerging technology. The first commercial device was produced twelve years later, in 1977, by Toshiba [3]. Since then, SAW device technology has improved drastically. A series of techniques and new materials have been developed to simplify the fabrication process, making such devices compact and with a low fabrication cost. Today, SAW devices represent a key technology in many fields, such as automotives, electronics, medical, aerospace, and defense [4,5,6,7,8,9]. Moreover, SAW resonators are widely used in sensing applications because of their unique features that enable them to be used as detectors in battery-less systems with remote wireless interrogation [10,11,12,13,14,15,16]. In this context, the operating principle of a SAW resonator is relatively simple. An acoustic wave travels through the surface of a piezoelectric material. Any stimulus or perturbation on the device surface (i.e., adsorbed gas molecules and liquids) may affect the propagation of the acoustic wave, thus altering the characteristic resonant frequency. Such variations can be measured with high-accuracy frequency estimation algorithms [17] and easily related to the physical quantity of interest [18]. SAW resonators have been successfully employed for temperature and humidity sensing [15,19,20,21], as pressure sensors [16,22,23], biosensors [24], and for VOC and gas sensing [25,26,27,28]. They have also been employed in harsh environments [14,29,30]. Among the features that characterize these devices, the temperature dependency plays an important role, especially in mobile and wireless communications, as well as in sensing applications. Therefore, analysis of the temperature-dependent characteristics becomes essential for enabling the use of commercial SAW devices in practical applications.

The present work is focused on the electrical characterization of a commercial SAW resonator over the temperature range between 0 °C and 100 °C. An equivalent-circuit model is extracted and validated in the entire range of the investigated temperature. Although SAW devices can be straightforwardly and successfully modeled using artificial neural networks (ANNs) [31,32,33], the equivalent-circuit model is easier to understand and is able to describe the physical behavior of the device. The SAW resonator selected in this work is a two-port packaged device with a nominal resonant frequency of 423.22 MHz. An equivalent-circuit model for a SAW resonator has already been developed and tested at room and cryogenic temperature [34,35,36]. It is worth noting that the topology of the equivalent-circuit model and the methodology for extracting the model elements are independent of the temperature condition; on the other hand, the behavior of the SAW device and the values of the model elements vary with changing temperature.

In this contribution, in order to extend the previous results, the model parameters are extracted and validated at higher temperatures, by heating the DUT up to 100 °C. The developed study, based on coupling an extensive temperature-dependent experimental characterization with equivalent-circuit modeling, enabled us to analyze in detail the SAW performance over the temperature range from 0 °C to 100 °C and to verify the accuracy and robustness of the modeling procedure, also at high-temperature conditions. Moreover, in order to further extend the previous studies [34,35,36], the extraction process is improved for a more accurate determination of the model parameters (i.e., the values of the equivalent-circuit elements). This improvement is accomplished by using a complex Lorentzian function to fit both real and imaginary parts of the short-circuit input and output admittances (i.e., *Y*_11_ and *Y*_22_) rather than a real Lorentzian function to fit only the real part of them [34,35,36], thereby allowing an improvement in the determination of the resonant parameters, which are used for the extraction of the equivalent-circuit elements.

The paper is organized as follows. The next section is divided into three subsections: the first part is devoted to a brief description of the measurement setup with special attention to the temperature control unit, whereas the second part is focused on the equivalent-circuit model and its analysis, and in the third subsection the methodology for the parameters extraction from the carried-out measurements is described. In Section 3, a comparison between the measurements and simulations is reported in order to verify the reliability of the employed equivalent-circuit model. Concluding remarks are given in the last section.

## 2. Materials and Methods

### 2.1. Measurement Setup

**Figure 1:**
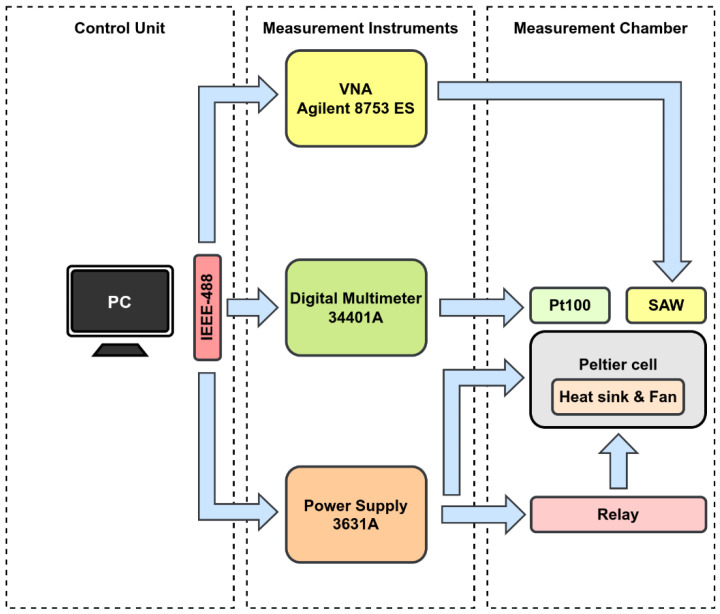
Schematic of the measurement setup.

The device studied in this work is a TO-39 packaged SAW resonator produced by Murata (Kyoto, Japan). Its manufacturer code is SAR423.2MDA30x80 [37], where SAW designates saw resonator [38], 423.2 M means that the resonant frequency is 423.2 MHz, DA means that the SAW has two ports, 3 means that the SAW is based on using the TO39-3 leaded package, 0 means that the specification designator is standard, x means that the substrate is made of quartz, and 80 indicates that the frequency tolerance is 80 kHz. The resonator was characterized in terms of admittance (*Y*-) parameters at different temperatures, ranging from 0 °C to 100 °C. To do this, a vector network analyzer (VNA) was used to acquire the scattering (*S*-) parameters that, using a python-based package, were converted into the *Y*- ones. The representation based on the admittance parameters was adopted for developing the reported study, since this representation is the most convenient for extracting straightforwardly and analytically the lumped equivalent-circuit elements. For the temperature characterization, a Peltier cell was exploited with its temperature control system unit. A schematic illustration of the measurement setup is reported in Figure 1. It consists of three main parts:

1. *Measurement chamber*: It includes a Peltier cell and a Pt100 thermoresistance, used for the temperature actuation and control, and a polarity-reversal relay. It is also equipped with all the connections needed by the device under test.
2. *Measurement instruments*: These include all the instrumentation involved in the measurement process. The Agilent E3631A power supply is used to provide the supply voltage to the Peltier cell and its cooling fan. The Agilent 34401A digital multimeter is used to measure the Pt100 resistance and convert it into a temperature unit as a feedback signal for the control system. Finally, the Agilent 8753ES VNA is employed for the electrical characterization, in terms of *Y-* parameters, of the microwave device under test.
3. *Control system unit*: Each measurement instrument included in the control chain loop is connected to a desktop PC that acts as a central control unit. It represents the third and last part of the measurement system. Through the IEEE 488.2 GPIB interface, the PC is able to set the right power supply for the Peltier cell and acquire the Pt100 resistance value and the *S-* parameters from the VNA. In addition, a custom-developed software allows not only the real-time data acquisition and processing, but also allows saving measurements on files for post-processing analysis.

**Figure 2:**
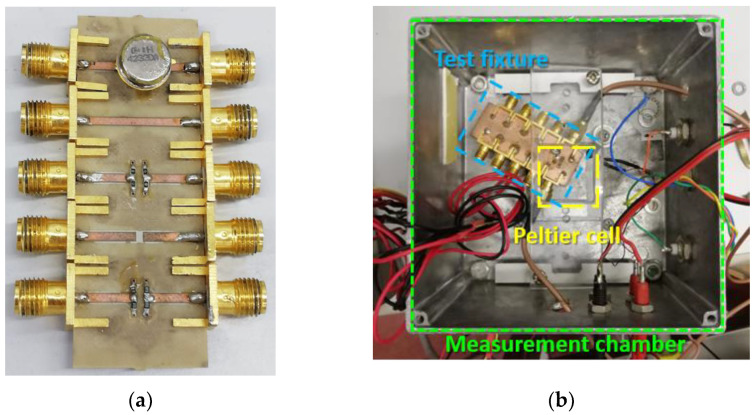
(**a**) DUT connected to the test fixture. (**b**) Measurement chamber with the test fixture mounted on the Peltier cell.

The SAW resonator was placed inside the measurement chamber and connected to the Agilent 8753ES VNA with coaxial cables for the acquisition of the S-parameters. A full two-port calibration was carried out using a short-open-load-through (SOLT) technique based on an ad hoc calibration kit [39]. The DUT and the measurement chamber with the test fixture mounted on the Peltier cell are depicted in Figure 2. The board was developed using an Arlon substrate by means of the Protomat S103 (by LPKF) rapid PCB prototyping machine.

The SAW resonator was placed in contact with the Peltier cell for the whole measurement process. The Peltier cell is a 90 W cell with a maximum temperature difference of 68 °C. Its dimensions are 40 × 40 mm^2^ with a thickness of 4 mm. The measuring system adopted can be used to carry out measurements at temperatures both above and below room temperature. In the latter case, the surface in contact with the SAW becomes cooler, while the opposite one tends to heat up accordingly. To prevent the cell from being damaged by excessive overheating and to improve its performance, a passive heat sink was placed in contact with the opposite side of the cell. This heat sink, placed outside the measurement chamber, allows the dispersion of the excess heat with the aid of a fan that, via software, automatically comes into operation when measurements at temperatures below the ambient temperature are carried out.

The temperature of the cell was controlled through a proportional–integral–derivative (PID) closed-loop control system. The feedback signal was provided by the Pt100 thermoresistance placed in contact with the Peltier cell, alongside the SAW under test. The custom-developed software, besides the implementation of the virtual PID control unit, is also used to acquire the *S-* parameters from the VNA at each selected temperature and is then able to convert them into *Y-* parameters and carry out the parameters extraction process for each temperature set point. A more detailed description of the extraction methodology is reported in Section 2.3.

### 2.2. Equivalent-Circuit Model

**Figure 3:**
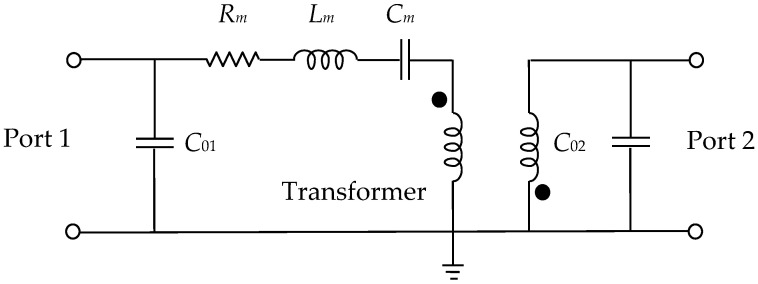
Electrical equivalent-circuit model for a two-port SAW resonator.

The equivalent-circuit topology, which is used to model the studied SAW resonator, is shown in Figure 3 [34,35,40,41]. This circuit is a two-port network, since the SAW under test is a two-port device. This two-port circuit can be simplified to a one-port network when investigating a one-port device. In such a case, both the experimental characterization and the model extraction procedure become simpler and faster.

The equivalent-circuit model in Figure 3 was successfully validated down to cryogenic temperatures [34], showing a good accuracy locally, near the resonant frequency. It is composed of the input/output shunt static capacitances, i.e., *C*_01_ and *C*_02_, by an *R_m_L_m_C_m_* series network, and by an ideal transformer. The model was developed considering the physical behavior of the SAW resonator: *C*_01_ and *C*_02_ represent the static capacitances at each port of the device; *R_m_*, *L_m_*, and *C_m_* are associated with the contributions of damping, inertia, and elasticity, respectively; and, finally, the transformer is meant to represent the conversion between mechanical and electrical energy. The transformer is considered to be ideal and its effect consists of producing a 180° phase shift in *Y*_21_ and *Y*_12_.

Although the equivalent-circuit elements provide only an approximate representation of what occurs in the device, they are physically meaningful parameters as they can be linked to the physical behavior of the SAW. Therefore, the variations in the equivalent-circuit elements are linked to the temperature-dependent variation in the physical behavior of the SAW. This implies that the analysis of the equivalent-circuit parameters versus the temperature allows for a better understanding of the underlying physics.

The analysis of the equivalent circuit depicted in Figure 3 shows that its resonant frequency *f_r_* can be expressed as:

(1) $$f_{r}=\frac{1}{2\pi \sqrt{L_{m}C_{m}}}$$

While the quality (*Q*) factor can be represented by the following expression:

(2) $$Q=\frac{2\pi f_{r}L_{m}}{R_{m}}$$

With reference to the definition of the nodal admittance matrix, the admittance (*Y-*) parameters for the selected equivalent circuit can be expressed as:

(3) $$Y_{11}=j\omega C_{01}+\frac{1}{R_{m}+j\left(\omega L_{m}-\frac{1}{\omega C_{m}}\right)}$$

(4) $$Y_{12\;}=Y_{21}=\frac{1}{R_{m}+j\left(\omega L_{m}-\frac{1}{\omega C_{m}}\right)}$$

(5) $$Y_{22}=j\omega C_{02}+\frac{1}{R_{m}+j\left(\omega L_{m}-\frac{1}{\omega C_{m}}\right)}$$

At resonance, the above parameters can be written as:

(6) $$Y_{11r}=j\omega C_{01}+\frac{1}{R_{m}}$$

(7) $$Y_{21r}=Y_{12r}=\frac{1}{R_{m}}$$

(8) $$Y_{22r}=j\omega C_{02}+\frac{1}{R_{m}}$$

Therefore, the values of *R_m_*, *C*_01_, and *C*_02_ can be straightforwardly extracted from Re(*Y*_11_), Im(*Y*_11_), and Im(*Y*_22_) at the resonance, respectively. The other parameters, i.e., *L_m_* and *C_m_*, can be calculated from the SAW resonant frequency and *Q* factor, the analytical expressions of which are reported in Equations (1) and (2), respectively. The description of the parameters’ extraction methodology is reported in the next section.

In first approximation, the SAW under test can be considered as a symmetrical device, with *Y*_11_ = *Y*_22_ and *Y*_21_ = *Y*_12_. This property was verified through the measurements carried out on the device. Only few deviations were observed on *Y*_11_ and *Y*_22_, while the reciprocity condition (i.e., *Y*_21_ = *Y*_12_) was completely fulfilled. For this reason, in this work only the *Y*_11_, *Y*_12_ and *Y*_22_ parameters are taken into account for the device characterization.

### 2.3. Equivalent-Circuit Parameter Extraction

Once the *Y-* parameters are derived from the VNA measurements, the values of *R_m_* and *C*_01_ can be straightforwardly extracted from Re(*Y*_11_) and Im(*Y*_11_), respectively. Considering the expressions previously reported of the *Y-* parameters at resonance (Equations (6)–(8)), the values of the concerned parameters can be calculated as:

(9) $$R_{m}=Re\left(\frac{1}{Y_{11r}}\right)$$

(10) $$C_{01}=Im\left(\frac{Y_{11r}}{2\pi f_{r}}\right)$$

Similarly, *C*_02_ can be estimated using Im(*Y*_22_):

(11) $$C_{02}=Im\left(\frac{Y_{22r}}{2\pi f_{r}}\right)$$

By measuring the *f_r_* and *Q* values from the measurements carried out on the SAW under test, the values for *L_m_* and *C_m_* can be derived from Equations (1) and (2), and can be written as:

(12) $$L_{m}=\frac{QR_{m}}{2\pi f_{r}}$$

(13) $$C_{m\;}=\frac{1}{{\left(2\pi f_{r}\right)}^{2}L_{m}}$$

The values Re(*Y*_11*r*_), Im(*Y*_11*r*_), Im(*Y*_22*r*_), *f_r_*, and *Q* are obtained from the measurements carried out on the device under test. In particular, *f_r_* is the frequency in which the Re(*Y*_11_) is maximum. Re(*Y*_11*r*_) and Im(*Y*_11*r*_) are the values of Re(*Y*_11_) and Im(*Y*_11_) at *f = f_r_*, respectively. Finally, *Q* is estimated from measurements as the ratio between the resonant frequency and the half-power bandwidth.

The determination of Re(*Y*_11*r*_), Im(*Y*_11*r*_), Im(*Y*_22*r*_), *f_r_*, and *Q* from the acquired measurements is not trivial. In particular, because of the presence of noise in measurements or because of limited points of the acquired spectrum, the estimation of such quantities could be inaccurate or, in other words, the measurement uncertainty may not fit the project requirements. In the literature, there are different strategies in order to increase the accuracy of the determination of *f_r_* and *Q_r_* [42,43,44]. For instance, a Lorentzian fitting can be performed on the acquired data points so that *f_r_*, *Q*, and the other parameters can be derived from the fitted equation analytically and with higher accuracy [45,46].

In this work, a Lorentzian function (*L*(*f*)) in the form of Equation (14) [46] was employed to perform a fitting of the *Y*_11_ and *Y*_22_ parameters.

(14) $$L\left(f\right)=\frac{a_{0}f}{f^{2}-f_{0}^{2}}$$

where *a*_0_ and *f*_0_ are two complex coefficients estimated by the selected best fit algorithm. The term *f*_0_ can be expressed as:

(15) $$f_{0}=f_{r}+jg$$

where *f_r_* is the SAW resonant frequency and 2*g* is the half-power bandwidth of the peak. For the selected Lorentzian function, the *Q* factor can be written as:

(16) $$Q=\frac{f_{r}}{2g}$$

In order to improve the quality of the fit, a background signal (*B*(*f*)) is usually included beside the Lorentzian peak [46]. It is a complex polynomial function expressed as:

(17) $$B\left(f\right)=\;\overset{N}{\underset{n=0}{\sum }}b_{n}{\left(f-f_{c}\right)}^{n}$$

where *N* is the polynomial order, *b_n_* are complex coefficients, and *f_c_* is a real quantity. In the present work, experimental measurements proved that a good description of the background signal is possible with *N =* 1. The final function used to model the generic admittance parameter (*Y_ij_*) is:

(18) $$Y_{ij}\left(f\right)=L\left(f\right)+B\left(f\right)=\;\frac{a_{0}f}{f^{2}-{\left(f_{r}+jg\right)}^{2}}+b_{1}\left(f-f_{c}\right)+b_{0}$$

A python script was developed to perform the fitting, estimate the required parameters (i.e., Re(*Y*_11*r*_), Im(*Y*_11*r*_), Im(*Y*_22*r*_), *f_r_*, and *Q*), and extract the equivalent-circuit model element values. For this purpose, the *scikit-rf* package and the non-linear least-squares minimization and curve-fitting (*lmfit*) library were used. *Scikit-rf* is an Open Source BSD-licensed package for python designed for RF/Microwave engineering. It was used to manage the acquired *S-* parameters, making all the conversions and calculations needed. This tool provides a modern and object-oriented library, very useful for data management in RF measurements. The *lmfit* library was used for the fitting procedure. It implements many optimization methods including the least square and the Levenberg–Marquardt method. The library is free and is released using an open source license.

The python script takes the acquired *S*-parameters as input, converts them into *Y-* ones, and performs the fitting using the Levenberg–Marquardt algorithm. Once an analytical expression for both *Y*_11_ and *Y*_22_ is obtained by the fitting procedure, the SAW resonant frequency is explicitly given by the coefficient *f_r_*, calculated by the best-fit algorithm. The *Q* factor is calculated using the Equation (16). The quantities Re(*Y*_11*r*_), Im(*Y*_11*r*_), and Im(*Y*_22*r*_) can be derived from the fitted functions at *f = f_r_*. The equivalent-circuit elements values are thus calculated using the Equations (9)–(13).

**Figure 4:**
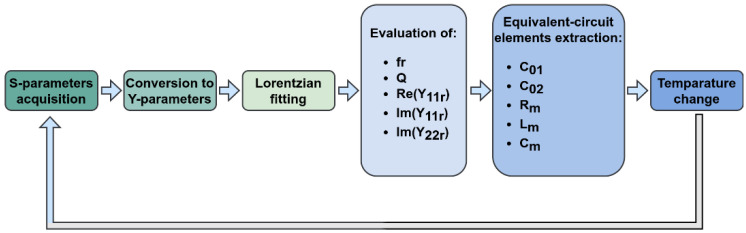
Diagram of the equivalent-circuit elements extraction process.

The extraction process is summarized in Figure 4.

**Figure 5:**
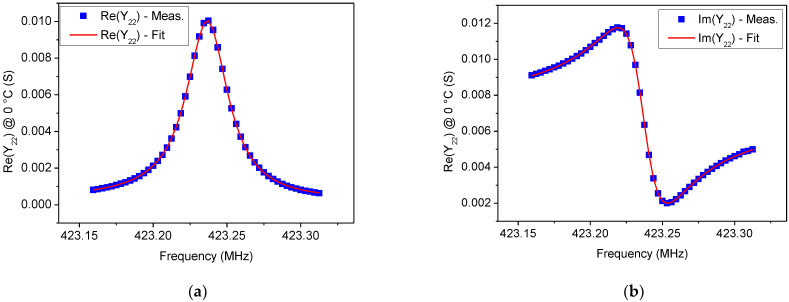
Comparison between measurements and Lorentzian fitting on the (**a**) Re(*Y*_22_) and (**b**) Im(*Y*_22_) for the tested SAW resonator at 0 °C.

**Figure 6:**
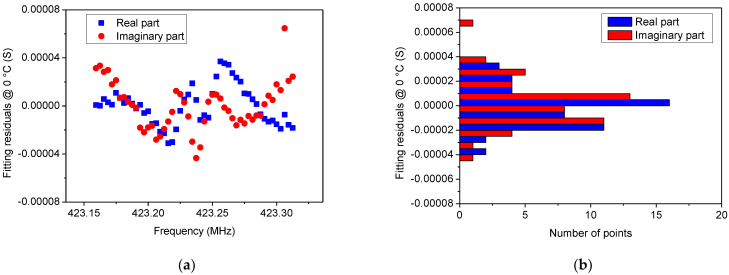
(**a**) Residuals of the Lorentzian fitting at 0 °C and (**b**) their probability distribution function that can be considered normal, thereby providing proof of good fitting.

**Figure 7:**
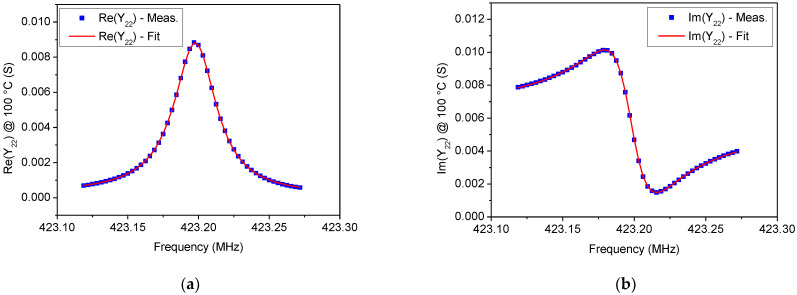
Comparison between measurements and Lorentzian fitting on the (**a**) Re(*Y*_22_) and (**b**) Im(*Y*_22_) for the tested SAW resonator at 100 °C.

**Figure 8:**
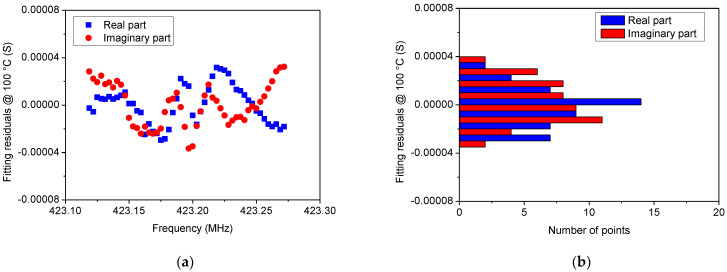
(**a**) Residuals of the Lorentzian fitting at 100 °C and (**b**) their probability distribution function that can be considered normal, thereby providing proof of good fitting.

In Figure 5, Figure 6, Figure 7 and Figure 8 a comparison between the measured *Y*_22_ and its fitted function is shown at 0 °C and 100 °C, respectively. Since Equation (14) describes a complex Lorentzian function, both real and imaginary parts are reported in Figure 5 and Figure 7, respectively. As can be observed, the Lorentzian function fits very well the acquired points of the resonant peak, as proof of the reliability of the fitting procedure. The residuals of the Lorentzian fitting and their probability distribution function at 0 °C and 100 °C are depicted in Figure 6 and Figure 8, respectively. Residuals are relatively small (<40 µS) for frequencies close to *f_r_*. The probability distribution functions can be considered normal as additional evidence of the goodness of fitting procedure.

By using the analytical expression for the *Y-* parameters, it is possible to estimate *f_r_*, *Q*, and the other parameters with higher accuracy.

## 3. Results and Discussion

The SAW resonator was tested in the temperature range from 0 °C to 100 °C with steps of 20 °C between each point. The temperature stabilization time was evaluated to be lower than 300 s. For each temperature set point, the *Y-* parameters were acquired by means of the VNA and the values of the equivalent-circuit lumped-elements were extracted from the measurements, as discussed in the previous section.

**Figure 9:**
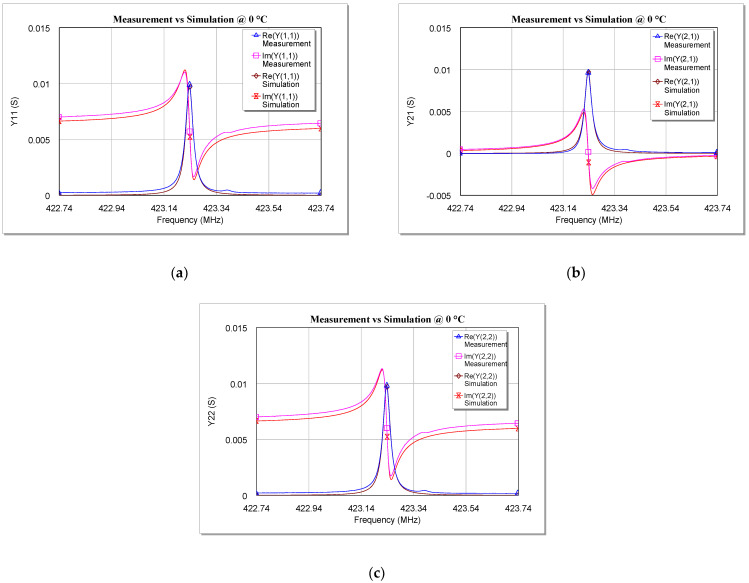
Comparison between measurements and simulations of the real and imaginary parts of (**a**) Y_11_, (**b**) Y_21_, and (**c**) Y_22_ versus the frequency for the tested SAW resonator at 0 °C.

**Figure 10:**
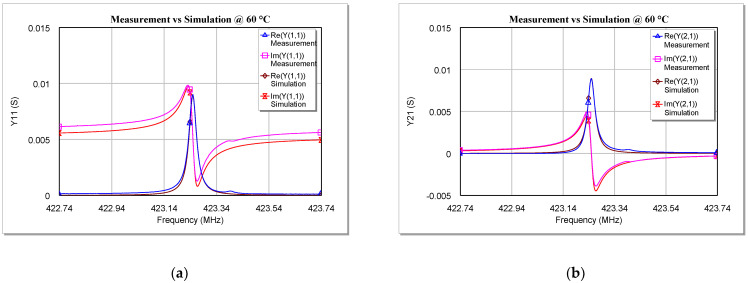

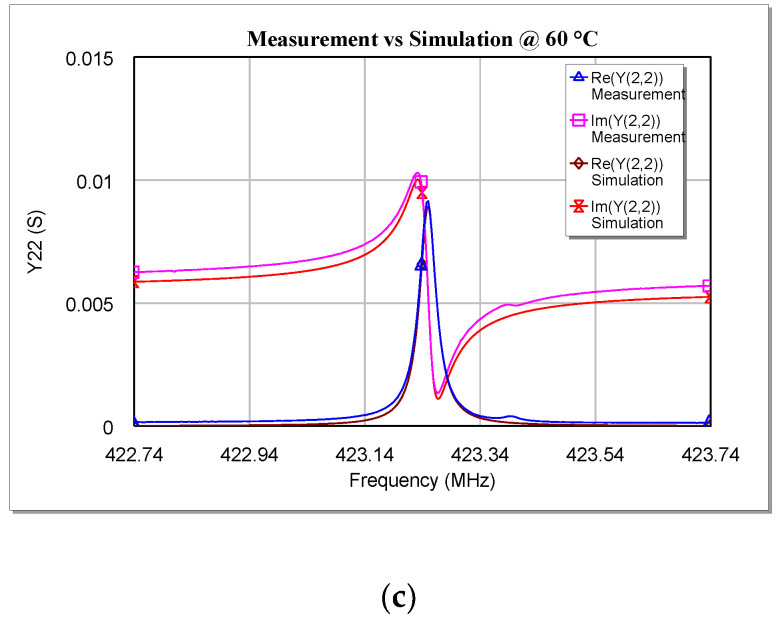
Comparison between measurements and simulations of the real and imaginary parts of (**a**) Y_11_, (**b**) Y_21_, and (**c**) Y_22_ versus the frequency for the tested SAW resonator at 60 °C.

**Figure 11:**
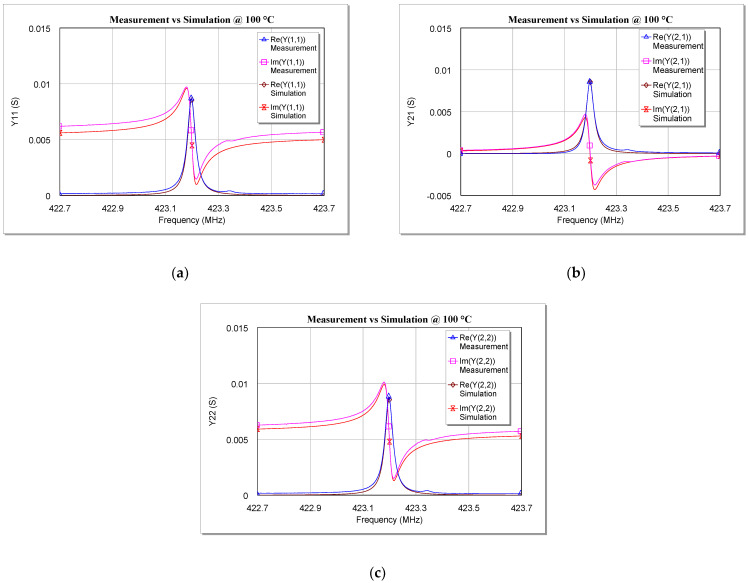
Comparison between measurements and simulations of the real and imaginary parts of (**a**) Y_11_, (**b**) Y_21_, and (**c**) Y_22_ versus the frequency for the tested SAW resonator at 100 °C.

Once the values of the five circuit elements were calculated, the equivalent circuit was implemented and simulated on computer-aided design (CAD) software in order to obtain the *Y-* parameters of the proposed model for each investigated temperature. These parameters were then compared with the measurements made on the SAW under test and the results of this comparison are shown in Figure 9, Figure 10 and Figure 11. For the sake of brevity, only the real and imaginary parts of *Y*_11_, *Y*_21_, and *Y*_22_ at 0 °C, 60 °C, and 100 °C are reported. Plots at other temperatures have been omitted here.

As can be seen from Figure 9, Figure 10 and Figure 11, the simulations are in very good agreement with the experimental results, especially for frequencies close to *f_r_*. This proves that the model validity is not limited to cryogenic temperatures [34], since the extracted model is also able to describe very well the behavior of a two-port SAW resonator at temperatures above the ambient temperature (at least up to 100 °C).

**Figure 12:**
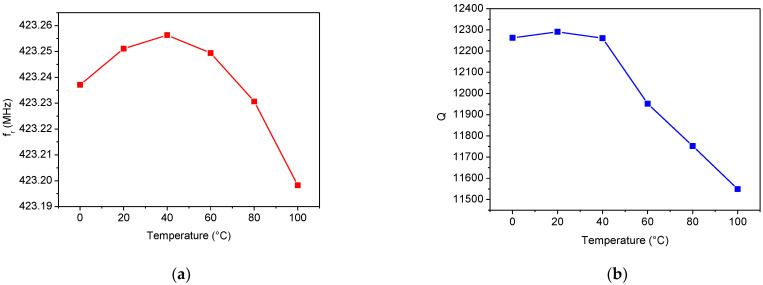
Temperature dependence of (**a**) the resonant frequency and (**b**) *Q* factor.

**Figure 13:**
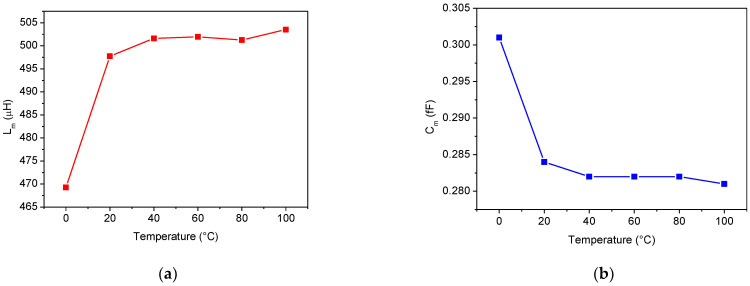

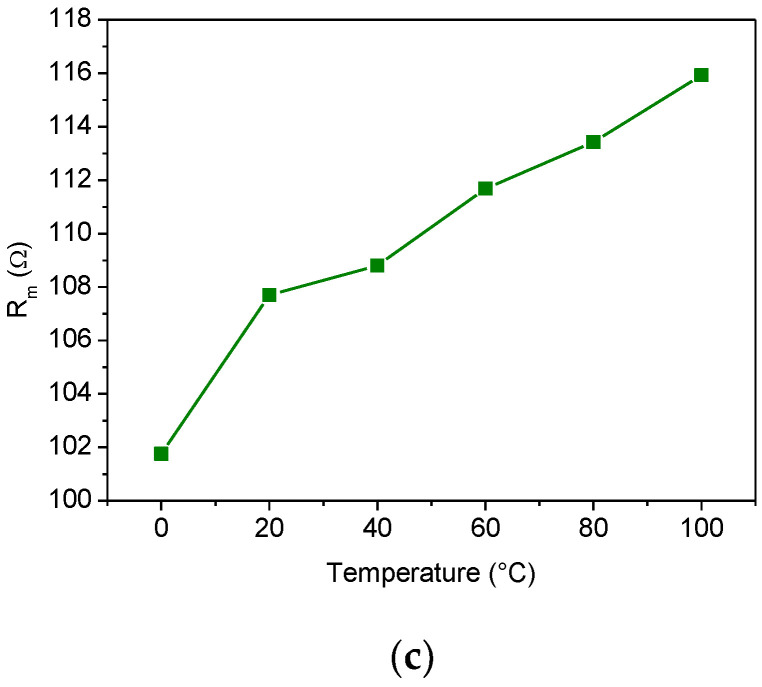
Temperature dependence of the motional elements: (**a**) *L_m_*, (**b**) *C_m_*, and (**c**) *R_m_*.

**Figure 14:**
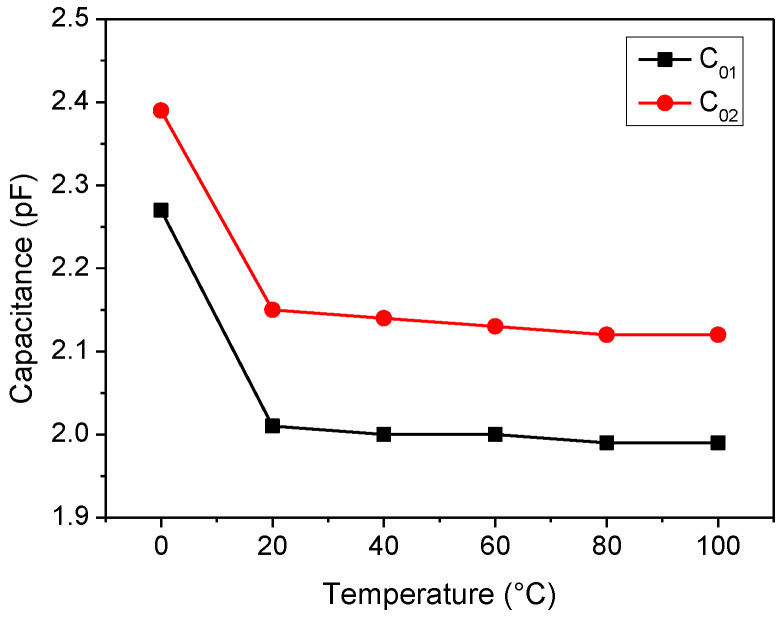
Temperature dependence of the input and output shunt static capacitances.

The temperature dependence of the extracted SAW parameters is reported in Figure 12, Figure 13 and Figure 14. In particular, Figure 12 shows the variations in *f_r_* and *Q* with the temperature. While the *Q* factor decreases almost linearly with increasing temperature, the resonant frequency has a parabolic trend with a maximum close to 40 °C. This behavior is not atypical, since it characterizes all the Murata SAW devices that belong to the SAR series [38].

The temperature dependence of the three lumped elements, *R_m_*, *L_m_*, and *C_m_*, is depicted in Figure 13. When the temperature increases, both the inductance and resistance values increase while the capacitance *C_m_* decreases. It is worth noting how the resistance value is related to the resonator *Q* factor. As described in Section 2.2, from a physical point of view, the resistance *R_m_* represents the damping effect. In other words, it is related to the resonator losses. As the temperature increases, the series resistance *R_m_* increases, thus lowering the resonator *Q* factor (see Figure 12a and Figure 13c).

Finally, the variation in the two input/output shunt static capacitances with the temperature is reported in Figure 14. In general, the extracted values of *C*_01_ are very close to those of *C*_02_. Except for an abrupt change in the values of the two capacitances when decreasing the temperature from 20 °C to 0 °C, *C*_01_ and *C*_02_ are almost constant and equal to 2.00 pF and 2.13 pF, respectively. Such behavior was not observed in [34], where a similar SAW was characterized using a cryogenic experimental system. In this case, the abrupt change of *C*_01_ and *C*_02_ at 0 °C may be ascribed to water vapor condensation on the SAW package affecting the equivalent parasitic capacitances of the case.

## 4. Conclusions

In the present article, a SAW resonator was characterized in terms of *Y-* parameters in the temperature range from 0 °C to 100 °C. A lumped-element equivalent circuit was employed to model the device under test over the investigated temperature range. Each element of the equivalent-circuit model was extracted from the measurement carried out on the physical device and circuit simulations were performed in order to evaluate the reliability of the proposed model. A good agreement between equivalent-circuit simulations and measurements was observed. The achieved results confirm the accuracy and robustness of the equivalent-circuit model, which is able to reproduce the SAW behavior under both cooled and heated conditions. The temperature dependence of each element of the proposed equivalent-circuit model (i.e., *R_m_*, *L_m_*, *C_m_*, *C*_01_, and *C*_02_) was described. Moreover, the variation in the SAW resonant frequency and *Q* factor with the temperature was reported and discussed. By increasing the temperature, the resonant frequency shows a parabolic trend and the *Q* factor decreases almost linearly.

The novelty brought by this investigation, compared with our previous studies, is twofold: extension of the model validation at a higher temperature by heating the tested SAW up to 100 °C and improvement of the model extraction by using a complex Lorentzian function to enable a more accurate determination of the model parameters.

As future work, the modeling procedure will be applied in the characterization and modeling of SAW gas sensors, using both devices already developed by Donato et al. [26,47] and new ones.

## Data Availability

Not applicable.
